# A Brief Review on the Pathological Role of Decreased Blood Flow Affected in Retinitis Pigmentosa

**DOI:** 10.1155/2018/3249064

**Published:** 2018-02-25

**Authors:** Yi Jing Yang, Jun Peng, Deng Ying, Qing Hua Peng

**Affiliations:** ^1^Hunan University of Chinese Medicine, Changsha, Hunan Province 410208, China; ^2^Department of Ophthalmology, The First Affiliated Hospital of Hunan University of Chinese Medicine, Changsha, Hunan Province 410007, China

## Abstract

Retinitis pigmentosa (RP) represents a clinically and genetically heterogeneous disease characterized by progressive photoreceptor loss. In recent years, research has been rarely made in blood flow affected in RP. The specific mechanism of blood flow affected in RP is not completely clear. A number of studies indicated that the decreased blood flow was related to RP. According to clinical observation and treatment experience, Chinese medicine considered that blood stasis runs throughout the RP disease progression, and the blood stasis corresponding to Chinese herbal medicine has a positive effect on the clinical treatment of RP. Therefore, we proposed that the decreased blood flow may participate in the lesion. In this article, we will review the findings on the decreased blood flow affected in RP from the perspective of modern medicine and Chinese medicine.

## 1. Introduction

Retinitis pigmentosa (RP) is a group of heterogeneous inherited photoreceptors' apoptosis and retinal degeneration disease characterized by constricted visual fields and photoreceptor cell dysfunction [1]. RP is the most common progressive and hereditary retinal degeneration, causing blindness and primarily affecting the peripheral retina; RP results from initiated defect in rod photoreceptors and invariably evokes secondary cone photoreceptor loss [2, 3]. Degenerated photoreceptors reduce the thickness of the outer layer in the retina, following retinal pigment epithelium (RPE) abnormal phagocytosis and involving choroid injury [4]. The retina receives its nutrients and oxygen from two separate circulations: retinal and choroidal circulation. The choroidal circulation is characterized by a high blood flow and a low arteriovenous difference in oxygen content. The retinal circulation in contrast is characterized by a low blood flow and high oxygen consumption. Both the retinal circulation and choroidal circulation contribute to the nutrition of retinal tissue. To this day, clinical findings on retinal vessels and blood flow suggest that microcirculatory changes may be drawn into the progression of RP, especially the loss of central visual function [5]. So far, there is rarely research on the mechanism of blood flow affecting the progressive process of RP, and the mechanism is not completely clear.

Chinese medicine is a style of traditional medicine built on a distinct foundation of more than at least 2500 years of Chinese medical practice and recently influenced by modern Western medicine. Chinese medicine diagnosis based on obtained symptoms aims to trace symptoms to patterns of an underlying disharmony; Chinese medicine treatment determination based on syndrome dialectic of underlying disharmony aims to regulate physical activity for balance. Both of the basic theories are essential principles in Chinese medicine [6, 7]. Therefore, the diagnoses and corresponding treatment in Chinese medicine indicate the occurrence and the process of the disease. In AD 992, RP was described as the high-altitude wind sparrow's vision in Chinese medicine. Chinese medicine considered that patterns of deficiency (including liver, heart, spleen, lung, or kidney deficiency) accompanied by the loss of nourishment in the eyes are the main etiology of RP. The most common etiology of RP is congenital deficiency, and blood flow cannot nourish the eyes. Based on the main etiology, by using corresponding Chinese herbal drugs, ophthalmologists of Chinese medicine have gained abundant experience to improve visual function [8]. Many clinical studies confirmed that most patients with RP are accompanied by symptoms of blood stasis. However, the current research of Chinese medicine on this syndrome is still at the clinical practice and needs further study.

## 2. Mechanisms of Cell Death in RP

To this day, molecular research has found and identified a huge number of mutations in genes associated with retinitis pigmentosa. However, molecular research suggests that different mutations involved in RP largely share a common functional or structural abnormality [9, 10]. The most common pathway of retinitis pigmentosa results from a primary defect in the rod photoreceptor cascade, a specific biochemical pathway that transduces light and leads to changes in photoreceptor cell polarization. Pathogenesis mutations in specific RP genes such as retinol binding and 3′,5′-cyclic-GMP phosphodiesterase activity would evidently interfere with rod dysfunction or structural isomerism and give rise to congenital night blindness. Subsequently, the death of rod photoreceptors is an invariable outcome and leads to the degeneration of RPE and cone photoreceptors [11]. RP mutations trigger alterations in function or structure involved in photoreceptor cell death, mainly owing to the following mechanisms of photoreceptor cell degeneration [12].

### 2.1. Apoptotic Mechanisms

Physiologically programmed cell death is believed to be triggered by genetic mutations, and the genetic mutation in RP is expressed exclusively in rod photoreceptors. However, cones can also undergo apoptosis following the loss of rods, due to the activation of apoptotic death in intracellular signaling and mediators in photoreceptors [13]. Presumably, the slow progressive cell death in response to a change in the retinal microenvironment is caused by the loss of rods [14]. Cell death leads to the deterioration of the cell survival environment and the abnormal physiological and biochemical characteristics consequent upon a decline in antioxidant defense and brings about vulnerability to cell death [15].

### 2.2. Light Damage Mechanisms

Light acts on rhodopsin triggers, the well-known visual transduction cascade, but can also induce cell damage and death through phototoxic mechanisms [16]; reactive oxygen and nitrogen species are generated when light affecting the retinoids leads to the ionization of biomolecules and release of all-*trans*-retinal. Afterwards, the excitation of all-*trans*-retinal generates oxygen enhancement and causes photooxidative damage [17]. Mutations in retinol metabolism and 3′,5′-cyclic-GMP phosphodiesterase activity affected the visual cycle and may block the recycling of all-*trans*-retinal to 11-*cis*-retinal, generating photooxidative damage and depositing the ocular pigment lipofuscin [18]. In addition, light exposure can accelerate the consumption of oxygen and the degeneration of photoreceptors [19].

### 2.3. Endoplasmic Reticulum Stress Mechanism

Disruptions of endoplasmic reticulum (ER) function result in the gathering of unfolded and misfolded proteins in the ER; cellular stress occurs while gathering unfolded or misfolded protein exceeds the capacity of ER and activates proapoptotic signaling pathways [20]. ER stress can develop in response to various stimuli including glucose starvation, hypoxia, defective protein secretion/degradation, and oxidative stress [21]. ER stress developed in response to oxidative stress leads to inflammatory disorders, phagocytic activation, excessive production of cytokines, and other internal conditions. These conditions usually stimulate local hypoperfusion, hypoxia, vascular endothelial cells injury, and other pathophysiological processes; these processes are related to the generation and mass release of free radicals [22].

### 2.4. Cilia Transport or Membrane Defect Mechanism

The choroidal capillary delivers to the outer aspect of the retina; the cilia connect membranes and are involved in the trafficking of substances between the inner segment and the outer region of retina [23]. RP mutations associated with the structure or function of cilia, expected to disrupt ion movements and consequent energy utilization to phototransduction, cause cell vulnerability till RPE phagocytosis and drag in the choroid [24, 25].

### 2.5. Metabolic Stress and mRNA Processing

In cells capable of apoptosis, metabolic stress is a potent activator of this form of cell death [26]. Mutations that affect numerous proteins are involved in intermediary metabolism or fatty acid metabolism and can facilitate retinal degeneration. Mutations in the ubiquitination of expressed pre-mRNA processing factors can exert a type of cellular stress similar to ER stress and can activate apoptotic signaling pathways [27]. Both metabolic stress and cellular stress can lead to microenvironmental stress.

Retinitis pigmentosa is a highly variable disorder; currently, more than 90 mutations of genes have been associated with RP, including genes for Leber congenital amaurosis (LCA) or for other maladies [28]. Most retinal degeneration is hastened by genetic mutations, and the photoreceptor cell death in the process of retinal degeneration is probably evoking the secondary photoreceptor loss. The mechanisms of photoreceptor cell death stimulate local hypoperfusion, hypoxia, and vascular endothelial cells injury and further affect blood flow. However, blood flow and environmental factors affecting the process of photoreceptor cell loss is not fully studied.

## 3. Decreased Blood Flow Affected in Retinitis Pigmentosa

Generally, modern medicine has confirmed that retinitis pigmentosa sets on with the degeneration of photoreceptors and is then involved with RPE and the choroid in the progression of cell death. Photoreceptors are located on the inner region of the retina where blood is mainly supplied by retinal circulation. Retinal circulation is characterized by a low level of flow and a high level of oxygen extraction [29]. The correct balance between inner retina blood supply and photoreceptor oxygen consumption is essential for photoreceptor cascade. Photoreceptor death occurs when photoreceptors commence their function and the supply of oxygen is below the level of consumption [30, 31]. Retinal circulation lacks autonomic innervation, shows an efficient autoregulation, and is mainly influenced by mediators released by endothelial cells and the surrounding retinal tissue. The progressive loss of photoreceptors is the main pathology of RP; throughout the progression, the retinal circulation undergoes extensive dysfunction due to the abnormal metabolic release by cell death, and further circulation dysfunction may evoke the secondary photoreceptor loss [32, 33].

The choroid is the vascular layer of the eye located on the outer region of the retina, and most of the nutrients in photoreceptors and RPE are dependent on the choroidal blood supply [34]. The choroid is an anatomical plexus abundant in blood vessels, whose capillaries are the most permeable in all microcirculation, and the choroidal blood flux is about 50 times that of the retinal circulation [35]. Because the capillaries do not completely distribute in the outer region of the retina, they cannot adjust the delivery of blood to the outer retina; on the other hand, the retina cannot store the delivery of blood. Therefore, the choroidal circulation is characterized by the inability to autoregulate [36]. Blood flow through the choroid at a high speed leading to the veins contains near-arterial levels of oxygen. The capillaries of the choroid, unlike those of the retina, readily leak plasma proteins, which diffuse to the photoreceptors to fulfill their prodigious metabolism [23]. These explain the low level of oxygen extraction and suggest that the choroidal circulation, besides supplying nutrients, may have more functions such as thermoregulation, volume buffer, and formation of aqueous humor [37, 38]. Reduced choroidal blood flow can disrupt retinal activities and lead to dysfunction in visual sensitivity [39].

Some clinical assessments and laboratory findings suggested the decreased blood flow involved in RP. Langham and Kramer [40] measured the intraocular pressure (IOP) pulse and concluded that choroidal ischemia is intently associated with visual loss and pigment cell degeneration in patients with retinitis pigmentosa. Peng et al. [41] inspected the blood flow from the aspect of rheoophalmography, blood rheology, and microcirculation and proposed that blood stasis is one of the pathogenic mechanisms. As gauged by laser Doppler velocimetry and the retinal function imaging, Beutelspacher et al. [42] have shown that retinal blood flow is decreased in patients with RP compared with that in age-matched controls and concluded that the attenuation of retinal vessels is a classical feature of RP. Turksever et al. [43] have shown that retinal venous oxygen saturation increases in patients with RP compared with that in the normal controls and suggest that the oxygen consumption of retina in patients with RP is decreased. The conclusion implicated that blood flow in patients with RP has increased viscosity and aggregation, and the blood supply gets diminished and becomes slow. Falsini et al. [5] further demonstrated that subfoveal blood flow is reduced in patients with RP and correlates with central cone function, as measured by laser Doppler flowmetry and assessed by focal electroretinograms (FERGs), respectively. Similarly, Zhang et al. [44] have found that retinal and choroid blood flow was significantly reduced in RP patients compared with that in age-matched controls and significantly correlated with a-wave amplitude by magnetic resonance imaging (MRI). Ayton et al. [45] have found that patients with RP have a thinner choroid than controls. They concluded that patients with poorer visual acuity or longer duration of symptoms tended to have thinner choroids, and information of choroidal thickness profile in RP is significant for research in the field of restorative vision. A recent study evaluated the macular blood flow changes in patients with RP, using laser speckle flowgraphy (LSFG) [46]. The results suggested that the reduction of macular blood flow occurs in an early phase of cone degeneration in RP, and decreased retina blood flow was associated with diminished visual sensitivity in patients with RP [46, 47]. A further study carried by Guadagni et al. [48] demonstrated that an enriched microenvironment can slow down inherited photoreceptor death, concomitantly decrease retinal inflammation, and create local conditions favorable to cell survival. These findings suggest that decreased blood flow affected the progress of RP. However, the specific mechanism of blood flow involved in cell degeneration remains unknown. It is generally thought that RP primarily sets on with rod cell defects and follows the attenuation of retinal and choroidal blood vessels. Research on the mouse model of RP showed molecular and morphological characteristics of nutritional starvation in dead cone cells, indicating insufficient nutrition, which is caused by the attenuation of retinal and choroidal blood vessels involved in cell death [49].

Chinese medicine is a unique system founded on long-term medical practice. Ancient Chinese doctors noted that all phenomena and activities in the body could be categorized into yin and yang (two opposites; interdependent, complementary, and exchangeable aspects of nature), and both a human being's psychological activity and physiological activity are states of integrated yin and yang in balance or harmony. Yin refers to the material compositions of the tissues and organs, and yang to functions. Besides, Chinese medicine theory believes that a human being is a whole and considers the five internal organs (liver, heart, spleen, lung, and kidney) as the core. There is a circulation of Qi (represents energy) and blood to connect the tissues and organs. A disease eventuates after a disturbance in the yin–yang balance, or abnormal flow of Qi or blood. Zheng is the description and analysis of the diagnosis of disease in Chinese medicine, a temporary state at one time and defined by symptoms and signs (most of the symbol states are obtained from the appearance of the tongue and the senses of pulse).

**Figure 1:**
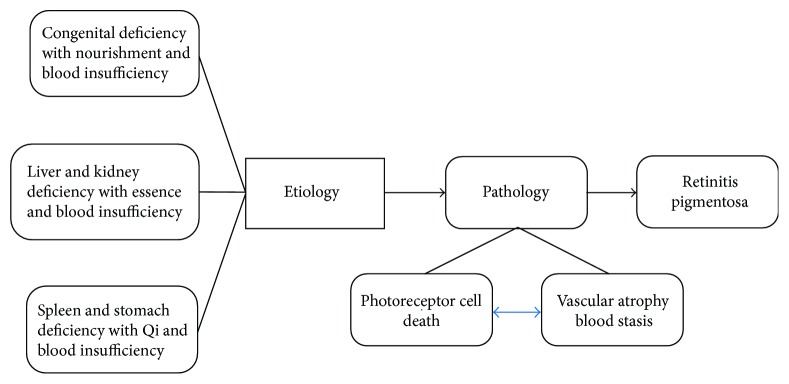
Blood stasis as effect of RP in Chinese medicine. In Chinese medicine, the main etiologies of RP include congenital deficiency, liver and kidney deficiency, and spleen and stomach deficiency; these etiologies are accompanied by nourishment and blood insufficiency, essence and blood insufficiency, and Qi and blood insufficiency in the eyes, respectively. The main etiologies may be independent or complex and can even be converted to each other. With the influence of these etiologies, patients of RP manifested vision impairment; vascular thinning, which the pathology process performs as photoreceptor death; and blood stasis.

Chinese medicine supposes the etiology of RP including congenital deficiency with nourishment and blood insufficiency in the eyes, liver and kidney deficiency with essence and blood insufficiency supply in the eyes, and spleen and stomach deficiency with Qi and blood insufficiency in the eyes (Figure 1). The etiology of RP in Chinese medicine is complex; the three etiologies may be independent or integrated in the patient; moreover, these etiologies can be converted to each other. Under the influence of the etiology, a patient of RP manifested vision impairment and vascular dystrophy. These clinical symptoms are conducted by pathology processes including photoreceptor death, vascular atrophy, and blood stasis. The changes in blood resulting in the loss of nourishment to the eyes and metabolites cannot be discharged. Consequently, the symptoms and signs of the spirit light whittle down (spirit light is related to Qi), nocturnal blindness occurs, and the visual field becomes narrow. These symptoms were described as the high-altitude wind sparrow's vision. In Chinese medicine based on changes in yin–yang (material and function), as well as the link of Qi and blood between the eyes and five internal organs, the dialectical Zheng of RP is classified as spleen-Qi deficiency, liver and kidney-yin deficiency, and kidney-yang deficiency (Table 1); these Zheng are associate with the symptoms of intraocular Qi and slowing down of blood flow, blood vessel atrophy, and dysfunction in vascular and neural remodeling. The diagnosis of RP in Chinese medicine is a guide for treating patients with a corresponding decoction (Table 1). For example, the Ming Mu Di Huang decoction (Chinese herbal drugs) and herb drugs of salvia can nourish the liver and the kidney and lead to invigoration of blood and improvement in visual function; thence, a formula of Ming Mu Di Huang decoction is specifically used to treat the liver and kidney-yin deficiency. In a similar way, You Gui Wan, a kind of Chinese medicine pill that can warmly invigorate the kidney yang, is specifically used to treat the kidney-yang deficiency. All the treatments mentioned above more or less involve the Chinese medicine activating blood dredge collateral (which promotes blood circulation by remove the meridian obstruction) to adapt the symptoms. Accordingly, Chinese medicine indicated that retinal blood flow change plays a critical role in the progressive degeneration of RP.

The blood flow of patients with RP originally presents viscosity and aggregation; the blood supply gets diminished and becomes slow, followed by vascular deterioration, eventually leading to vascular atrophy. On the one hand, studies indicated microenvironmental changes following rod degeneration, such as inflammation and oxidation [49]; on the other hand, environmental enrichment can reduce the death of photoreceptors and lead to the survival of retinal cells [48]. Clinical studies have shown that calcium blocker drugs can increase blood flow in the choroid and ameliorate the loss of central visual sensitivity in patients with RP [50]. Treatment determination based on symptoms and signs is an essential principle in Chinese medicine; the Ming Mu Di Huang decoction is specifically used to treat liver and kidney-yin deficiency and has obtained a positive effect on patients whose diagnoses were high-altitude wind sparrow's vision with kidney-yin deficiency and essence and blood insufficiency supply in eyes; in addition, as salvia (a kind of herb activating blood dredge collateral belonging to Chinese medicine) can accelerate blood circulation, the amount of salvia can improve the efficacy [51]. Chinese herbal drugs based on amelioration of retinal-choroidal blood stasis can promote blood circulation and improve vascular deterioration. What is more, Chinese herbal drugs can provide nutrition and protect factors to preserve photoreceptor cells and vascular cells. Ding Shuhua et al. [52] used the Yeming formula to treat retinitis pigmentosa and compared vision, visual acuity, and flash electroretinography in 92 eyes of 46 patients before and after treatment. The results showed 76 of 92 eyes (82.6%) had improved vision, micro-b wave appeared in 44 of 66 eyes' extinction types by flash electroretinogram, and the visual acuity of 54 of 66 eyes showed increased visual acuity. Dan [53] randomly divided 83 eyes of 42 patients with retinitis pigmentosa into the treatment group and control group. The treatment group was treated with Ming Mu Di Huang decoction, and the control group was given oral lutein and vitamin A treatment. After treatment, the visual acuity and visual field of patients have significantly improved. The effective rate of visual acuity and visual field in the treatment group is 80.48% and 78.04%, respectively, compared with those of the control group of 24.39% and 26.83%. Acupuncture has been applied as a therapeutic technique in Chinese medicine. By promoting blood circulation with acupuncture, a patient with RP showed vision and visual field distribution, and the ERG-b waves were significantly improved in 13 of 15 cases with RP [54]. Chinese medicine improves retinal nutrition and metabolism by ameliorating microcirculation and ischemic conditions of retinitis pigmentosa, further improving visual function and retarding the progression of retinitis pigmentosa.

In summary, the present studies and clinical treatments demonstrated that decreased blood flow is associated with the progression of photoreceptor degeneration. Improving the decreased blood flow and enriching the environment of retina are effective in ameliorating the degeneration of photoreceptors. However, the specific mechanisms of decreased blood flow involved in RP need to be further elucidated. Chinese medicine has a distinguished strategy to investigate the mechanism and therapy of RP. Combined with Chinese medicine, the distinguished perspective would help us understand the process of cell degeneration and the process of development in RP. The integration of Chinese medicine and modern medicine would widely promote studies on RP.

## Figures and Tables

**Table 1:** The therapy of RP in Chinese medicine.

Dialectic Zheng	Spleen-Qi deficiency	Liver and kidney-yin deficiency	Kidney-yang deficiency
Symbol state	Tongue, white and thin coating; pulse, weak	Tongue, red and thin coating; pulse, tenuous and high frequency	Tongue, pale and thin coating; pulse, sinking
Therapy	Invigorating the spleen and replenishing Qi	Nourishing the liver and kidney	Warmly invigorating the kidney yang
Prescription	Buzhong Yiqi decoction and Chinese medicine activating blood dredge collateral	Ming Mu Di Huang decoction and Chinese medicine activating blood dredge collateral	Yeming formula and Chinese medicine activating blood dredge collateral

Activating blood dredge collateral promotes blood circulation by removal of meridian obstruction.

## Abbreviations

RP: Retinitis pigmentosa
RPE: Retinal pigment epithelium
GMP: Guanosine monophosphate
ER: Endoplasmic reticulum
mRNA: Messenger RNA
LCA: Leber congenital amaurosis
IOP: Intraocular pressure
FERGs: Focal electroretinograms
MRI: Magnetic resonance imaging
LSFG: Laser speckle flowgraphy.
